# Redescending Stomach: A Rare and Potentially Lethal Complication of Gastric Herniation

**DOI:** 10.5334/jbsr.3448

**Published:** 2024-02-28

**Authors:** Sam Verrept, Mathieu Lefere, Yves De Bruecker

**Affiliations:** 1 Radiology Department, Imeldaziekenhuis, Bonheiden, Belgium; 2 Radiology Department, Imeldaziekenhuis, Bonheiden, Belgium; 3 Radiology Department, Imeldaziekenhuis, Bonheiden, Belgium

**Keywords:** gastric herniation, fundus redescent, strangulation, gastric pneumatosis, obstruction, ischemia

## Abstract

Large gastric hernias are common and usually cause minor symptoms. Rarely, complete intrathoracic herniation of the stomach is complicated by strangulation. The underlying mechanism can be gastric volvulus or the less recognized phenomenon of gastric fundus redescent. We describe a case where this rare but potentially lethal complication of gastric herniation is present. Additionally, we show that gastric pneumatosis, a sign associated with ischemia, can be initially visualized on a plain chest radiograph in this setting.

*Teaching point:* Redescent of the fundus is a possible, but unrecognized cause of gastric strangulation in intrathoracic stomachs.

## Case History

**Figure 1:**
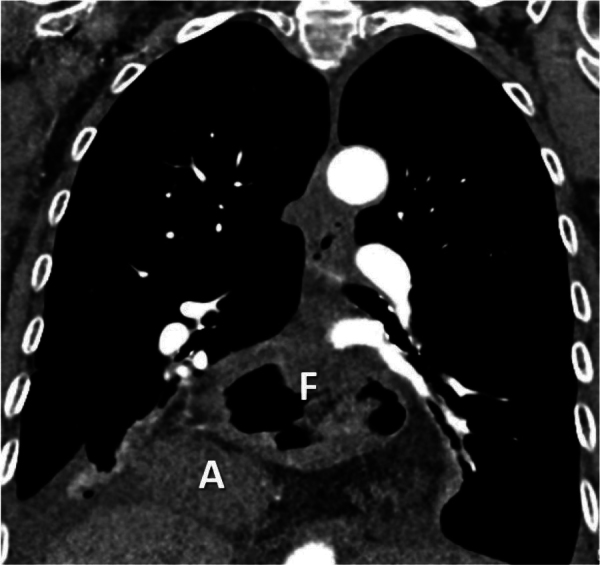
Prior chest CT illustrating the complete intrathoracic herniation of the stomach, with the fundus (F) located above the antrum (A).

**Figure 2:**
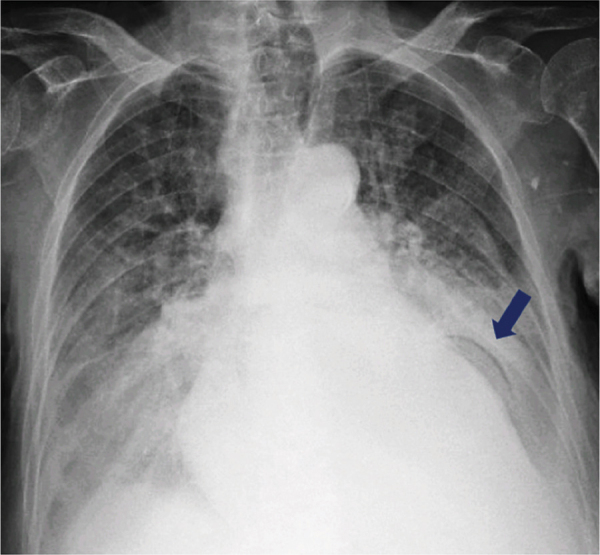
Follow-up chest radiograph showing gastric pneumatosis as a linear lucency outlining the circumference of the intrathoracic stomach (arrow).

**Figure 3:**
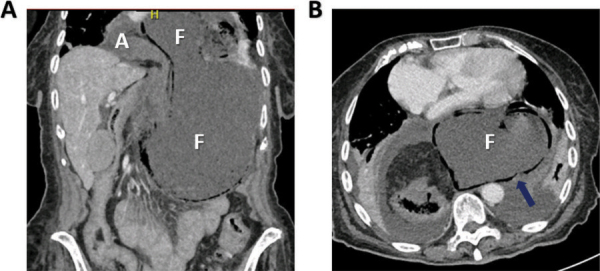
**(A)** Coronal reconstruction of the CT scan illustrating the probable mechanism of gastric fundus redescent in the intrathoracic herniated stomach: Migration of the fundus (F) through the hernial ring results in constriction and fundal dilatation, leading to crowding of the hernial orifice and strangulation. The gastric antrum (A) is still situated intrathoracically. **(B)** The presence of gastric pneumatosis is apparent in this ischemic context (arrow). The dilated stomach caused compression of the heart and lung parenchyma.

A 95-year-old female patient presented to the emergency department with sudden-onset epigastric pain and postprandial vomitus. She had a history of complete intrathoracic herniation of the stomach (Figure 1). Biochemistry showed leukocytosis with elevated lactate levels. An initial chest radiograph showed an intrathoracic stomach with adjacent hypoventilation zones. On a follow-up chest radiograph, linear lucencies outlining the circumference of the intrathoracic stomach appeared (Figure 2). An urgent contrast-enhanced computed tomography (CT) scan was performed. This showed intrathoracic herniation of the stomach and the right side of the colon transversum through the diaphragmatic hiatus. The gastric fundus had redescended into the abdomen and appeared grossly dilated, with prominent intramural gas (Figure 3). Given her old age, comorbidities, and signs of advanced ischemia on CT, the patient was not considered for surgery. She was administered the best supportive care and passed away within 24 hours of admission.

## Comments

Herniation of the stomach into the thoracic cavity begins with the development of either a paraoesophageal or sliding-type gastric hernia, most often secondary to age-related weakening of the diaphragm and/or increased intra-abdominal pressure. Upward migration of the stomach is limited by the retroperitoneal attachment beyond the proximal duodenum and by the subdiaphragmatic fixation of the distal esophagus. Because of these anatomic fixation points, it is possible for rotation (volvulus) of the stomach to occur with progressive intrathoracic herniation [1]. Two types of gastric volvulus have been described: organoaxial rotation, in which the stomach rotates around its long axis, and the less common mesenteroaxial rotation, in which it rotates around its short axis [2]. In general, gastric volvulus is a chronic condition with patients experiencing vague abdominal discomfort. Sometimes it can be complicated by strangulation, usually in the organoaxial subtype [3].

**Figure 4:**
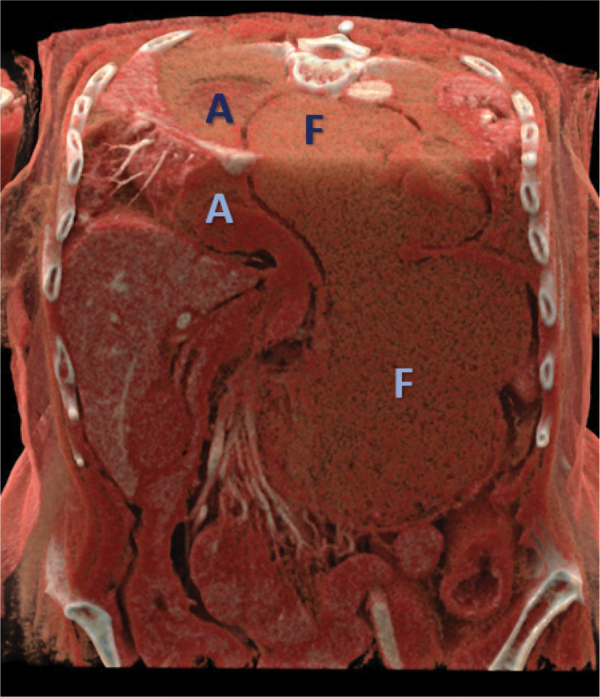
Cinematic rendering providing an overview of the descended and dilated gastric fundus (F) and intrathoracic gastric antrum (A), relative to the crowded hiatal orifice.

A less recognized cause of acute gastric strangulation is the redescent of the gastric fundus through the hernial ring. This is probably triggered by a sudden increase in intrathoracic pressure, causing the fundus to migrate back into the abdominal cavity [4]. As a result, the fundus cannot drain its contents through the constricted hernia neck into the supradiaphragmatic antrum. Food and fluid accumulate in the progressively dilating fundus, which leads to further crowding of the hernial orifice, eventually resulting in vascular strangulation of the distal stomach [1], [4]. This phenomenon is illustrated by the consecutive abdominal CT scans and cinematic rendering of this case (Figures 1, 3, and 4). A redescending gastric fundus as the cause of strangulation has been described in fluoroscopic studies in an article from 1976 [1]. Interestingly, since then, only one small case series seems to have been published on the subject [4].

Of note, the gastric strangulation in this case was also associated with pneumatosis, initially visualized on a chest radiograph. The presence of gas within the wall of the digestive tract is hypothesized to be caused by luminal gas diffusion and/or direct mural invasion of gas-producing bacteria in the context of increased mucosal permeability due to mucosal injury [5]. In bowel wall necrosis, pneumatosis is more often associated with acute mesenteric ischemia (29%–59%) than with strangulated bowel obstruction (7%), making the presentation of pneumatosis in gastric strangulation a rather infrequent CT finding [6]. It should also be noted that, in general, pneumatosis is a nonspecific sign for gastric ischemia that often has a benign, non-ischemic etiology [2].

This case illustrates that redescent of the gastric fundus is a potential mechanism leading to strangulation, and that represents a highly specific, yet rare complication of large gastric hernia.
